# Fuzzy Logic-Based Guaranteed Lifetime Protocol for Real-Time Wireless Sensor Networks

**DOI:** 10.3390/s150820373

**Published:** 2015-08-18

**Authors:** Babar Shah, Farkhund Iqbal, Ali Abbas, Ki-Il Kim

**Affiliations:** 1 College of Technological Innovation, Zayed University, Abu Dhabi 19282, UAE; E-Mails: babar.shah@zu.ac.ae (B.S.); farkhund.iqbal@zu.ac.ae (F.I.); 2 Department of Informatics, Research Center for Aerospace Parts Technology, Gyeongsang National University, GyeongNam 52828, Korea; E-Mail: aliabbas_ms@hotmail.com

**Keywords:** wireless sensor networks, real-time, fuzzy logic, routing

## Abstract

Few techniques for guaranteeing a network lifetime have been proposed despite its great impact on network management. Moreover, since the existing schemes are mostly dependent on the combination of disparate parameters, they do not provide additional services, such as real-time communications and balanced energy consumption among sensor nodes; thus, the adaptability problems remain unresolved among nodes in wireless sensor networks (WSNs). To solve these problems, we propose a novel fuzzy logic model to provide real-time communication in a guaranteed WSN lifetime. The proposed fuzzy logic controller accepts the input descriptors energy, time and velocity to determine each node’s role for the next duration and the next hop relay node for real-time packets. Through the simulation results, we verified that both the guaranteed network’s lifetime and real-time delivery are efficiently ensured by the new fuzzy logic model. In more detail, the above-mentioned two performance metrics are improved up to 8%, as compared to our previous work, and 14% compared to existing schemes, respectively.

## 1. Introduction

In wireless sensor networks (WSNs), the energy constraint is a very crucial issue, as sensor nodes are usually operated on limited and irreplaceable battery power. Thus, most research works have focused on how to prolong network lifetime by reducing the energy consumption of sensor nodes [1,2,3]. For the point of network management, a predictable, as well as certain guaranteed network lifetime is preferred over an unpredictable long lifetime. For example, if the network lifetime is guaranteed by the pre-determined time, many deployment problems can be easily solved [4]. However, research works for guaranteeing network lifetime have rarely been studied despite their impact on network management. Furthermore, since these existing schemes did not concern specific applications, they therefore suffer from adaptability problems in real deployment. Most WSN applications are required to meet specific requirements. In emergency WSN applications, such as radiation monitoring, security surveillance and fire detection, real-time data delivery is essential. In such applications, it is more desirable to continually accomplish a given task of the sensor nodes until the given network lifetime is expired. If an unpredictable lifetime is assumed, it is very hard to bound a delay deadline while considering energy constraints in WSNs. For this reason, a guaranteed lifetime for real-time applications in WSNs is a more appropriate deployment model than prolonging network lifetime.

In our previous work [5], we balanced the active and sleep times of each node to meet real-time requirements in a guaranteed network lifetime. However, due to the conflicting situations and imprecisions in the data, the problem of balanced energy consumption among sensor nodes remains unsolved. To design a guaranteed WSN lifetime protocol to provide support for real-time applications, the communication protocols must adjust their routing performance based on the packet deadline. However, it is not an easy task to simultaneously achieve guaranteed lifetime and real-time service in WSNs, because it is not feasible to describe them accurately by mathematical models. To address this problem, fuzzy logic can be an alternative model that has the potential for dealing with conflicting situations and imprecision in data using heuristic human reasoning without the need of complex mathematical models [6]. Furthermore, general fuzzy logic-based solutions are computationally quick, require modest resources and are able to drive effective and efficient results on a real-time basis [7].

Based on the aforementioned research motivations, we extend our previous work [5] in this paper by adding fuzzy controller algorithms to guarantee a pre-configured lifetime *α*, while achieving the real-time data delivery requirement. A fuzzy system is the combination of both systems and uncertainty models to configure the active/sleep mode of each sensor node. Through a fuzzy controller, an active/sleep scheduling is defined as an energy-efficient way to set a sensor’s mode in each round. In more detail, the proposed scheme is achieving its objective by enabling each sensor node to consume energy approximately at the optimal energy consumption rate in order to guarantee a pre-configured lifetime. Consequently, since the number of active sensors may not be enough to satisfy two requirements at once in the network for the whole period of operations, we adopt a new three-tier node selection mechanism to further enhance our previous scheme. In the first tier, the sensing nodes are selected, followed by the selection of normal transmission range (NTR) nodes in the second tier and the selection of extended transmission range (ETR) nodes in the third tier. We consider real-time constraints by forcing a power-aware transmission in ETR nodes selected in the third tier. To forward real-time data packets through third-tier ETR relay nodes, the fuzzy controller accepts energy and velocity as input descriptors for the third-tier ETR relay node selection. To summarize, the objective of this adaption is to not only decrease the delay, but also to improve the percentage of successful real-time packets in a guaranteed WSN lifetime. Simulation results are given to prove the effectiveness of the proposed scheme in the aforementioned performance metrics.

The rest of this paper is organized as follows: In Section 2, we discuss the related works. The proposed scheme and analysis of the algorithms for a guaranteed network lifetime and real-time communications are explained in Section 3 and Section 4. In Section 5, the simulation experiments that evaluate this scheme are presented. Finally, Section 5 concludes with a critical summary and some direction for future work.

## 2. Related Works

To better understand the related works and research motivation of the proposed scheme, first we must define the guaranteed networks lifetime. In this paper, we use the term guaranteed lifetime to describe the time in which a wireless sensor network must perform given operations before it is expired. As for network lifetime, much of the research literature has studied this in the last few decades. In this section, we briefly describe the related research works for energy consumption and network lifetime.

Several techniques have been suggested to reduce the energy consumption of sensors and to prolong network lifetime. As a centralized approach for scheduling and prolonging a WSN lifetime, the concept of utilizing artificial intelligence (AI) or computational intelligence (CI) techniques to support the decision-making process is widely used in the recent literature to obtain more efficient WSN algorithms. Several protocols applied AI or CI techniques to design energy-efficient WSNs and routing algorithms [8]. Such techniques emphasized the efficiency and performance of WSN routing protocols by merging sensed data obtained from nodes and their communication in order to improve network performance. Furthermore, these approaches require a large number of messages to be exchanged to improve the global network performance. Other AI- and CI-based protocols require high computational capabilities and a complete knowledge of the WSN prior to deploying the sensor nodes.

On the other hand, fuzzy logic-based approaches performed very well in energy efficient routing and clustering heuristics in WSNs. In [9], a fuzzy logic approach is used to select a cluster head by considering energy and distance to efficiently route data in WSNs. Another fuzzy logic-based energy-efficient algorithm, (Cluster Head Election mechanism using Fuzzy logic (CHEF) [10], selected cluster heads in a distributed way over energy and distance as input descriptors. Among other researchers, the energy-aware distributed dynamic clustering protocol [11] is proposed through fuzzy logic to consider the cost and distance of a node in the selection of a cluster head. Fuzzy routing algorithms (FML) were proposed in [12] to maximize lifetime routing problem in WSNs. FML results showed that the use of fuzzy functions and operators provides a promising direction for devising efficient solutions to numerous related energy-aware routing problems in WSNs. Although all of these schemes showed better performance than other mathematical models, they were limited only to energy-efficient routing in WSNs. In WSNs, the approaches based on fuzzy logic can be easily found for efficient energy utilization and routing [7,8,9,10,11,12,13]. However, due to the unbalanced remaining energy schemes among nodes, the existing techniques of maximizing WSN lifetime cannot be directly applied to a guaranteed lifetime.

In addition to minimizing the energy usage to extend the WSN lifetime, few research works have been proposed guaranteeing a pre-configured WSN lifetime. An integer linear programming (ILP)-based model is proposed in [14] to design a sensor network with guaranteed lifetime by periodically determining the minimum number of relay nodes as cluster heads. In [15], the pre-configured network lifetime is guaranteed by the duty cycle of each node. However, this algorithm relied on traffic load to reduce end-to-end latency and did not monitor the energies among nodes to provide provable guarantees on the network lifetime. Differently from the two above-mentioned approaches, adjustable battery packs were used in [16] to precisely determine the energy budget at deployment to guarantee the WSN lifetime. However, an extra introduced lifetime variable created a large amount of communication overhead. Similarly, a new cost function is introduced to balance the load over each link and to reduce the queuing delay to guarantee network lifetime [17]. Moreover, based on the residual energies of all of the nodes in the network, a directed acyclic graph is proposed in [18] to guarantee the WSN lifetime. A distributed approach is used in [19] to schedule nodes to increase the network lifetime and to decrease overall energy consumption by turning off some redundant nodes. Even though all of these approaches evaluated the guaranteed lifetime by a heuristic solution using simulation, they failed to concede balanced energy consumption among sensor nodes. Moreover, most of them generated a profusion of communication overhead. In addition, all existing schemes ignored real deployment for different applications, but targeted only one direction, *i.e.*, guaranteeing a pre-configured network lifetime or maximizing network lifetime.

When it comes to applications, real-time communication becomes one of the essential components for real deployment. Most sensor applications are very sensitive in terms of delay; many WSN applications need to resolve the inherent conflict between energy-efficient communication and desired end-to-end communication delay [20,21,22,23]. Both SPEED [20] and Multipath Multi-SPEED (MMSPEED) [21] are more promising QoS-based routing protocols that provide a soft end-to-end deadline for real-time packets in sensor networks. These protocols use a geographic forwarding mechanism to route packets to the sink. To guarantee a real-time packet, SPEED ensures a network-wide speed of packet delivery, while MMSPEED uses multiple speed layers. The soft real-time power-aware RACE protocol [22] effectively balances the load by using non-deterministic forwarding. A multi-path routing algorithm, QoS and Energy aware Multi-PAth Routing (QEMPAR) [23], addresses QoS in terms of timeliness and energy efficiency for real-time applications in wireless sensor networks. However, these protocols have the following two shortcomings: (1) the time delay caused by the channel is ignored in almost all existing real-time schemes in WSNs; and (2) high time complexity in these schemes makes them unsuitable for computing on resource-constrained environments.

As a result, as far as the authors know, there is no particular answer for guaranteeing the pre-configured network lifetime *α* while maintaining real-time packet requirements at the same time in WSNs, with the exception of our previous work.

## 3. Proposed Scheme for Guaranteeing Network Lifetime

The objective of the network lifetime guaranteeing problem is to ensure the network lifetime at least as long as the user or application demands. Since the network lifetime comes to an end when any node collapses its energy, the best way to satisfy the WSN’s user or application demands for a pre-configured network lifetime is to distribute energy consumption adequately among sensor nodes. In this article, we propose a guaranteed network lifetime mechanism to achieve user- or application-specified network lifetime through a novel algorithm based on a fuzzy logic controller. We assume that the user- or application-specified network lifetime is a parameter known or given at the beginning of network deployment.

We primarily focus on how to guarantee the previously-determined or pre-configured wireless sensor network’s lifetime. In order to achieve this, we endorse a strategy where each node consumes energy approximately at the optimal energy consumption rate $\frac{\beta }{\alpha }$, where *α* denotes the pre-configured network lifetime and *β* represents the sensor node initial energy. With this assumption, the pre-configured lifetime *α* is guaranteed if each node consumes energy at the proposed optimal rate $\frac{\beta }{\alpha }$. In order to enforce each sensor node to consume its energy approximately at the optimal consumption rate, we adopted a centralized scheme to schedule sensor nodes in active/sleep mode in each round. We are also aiming to achieve real-time service while guaranteeing the pre-configured lifetime; therefore, we employ a power-aware extending transmission range mechanism. The proposed scheme involves round-based operations where the tasks of each round are completed in two different phases. A detailed description of the mentioned phases is available in our previous research [1].

## 4. Fuzzy Logic Model

**Figure 1:**
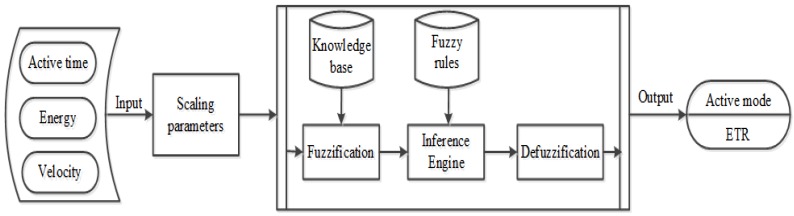
Fuzzy logic controller structure.

Instead of classical linear controllers or mathematical models, we introduce a fuzzy logic-based controller in this article due to its simplicity, clarity and suitability with WSN applications. Fuzzy systems are very useful in situations involving a highly complex system whose behaviors are not well understood and in situations where an approximate, but fast, solution is warranted [24]. Since guaranteed lifetime and real-time communication in wireless sensor networks are dealing with both kinds of situations, therefore a fuzzy system can be an ideal possible solution. To deploy it in the real world, the proposed fuzzy controller algorithm must be installed on both sink and sensor nodes in the network. The fuzzy controller in the sink node accepts input descriptors, energy and active time, for active/sleep scheduling. Similarly, fuzzy controllers in sensor nodes use controllable inputs of energy and velocity for the selection of the next hop relay node. The proposed fuzzy system consists of three parts; fuzzification, inference engine and defuzzification, as shown in Figure 1, along with fuzzy system factors. Through the proposed fuzzy logic, fuzzy logic determines a node role for the next round, either active or sleep, by taking the active time, velocity and energy on the node. Upon identifying the possible active nodes, a node is designated as either ETR or NTR. Therefore, each node’s rule for next round is determined by the proposed fuzzy logic consequently.

### 4.1. Fuzzification

In fuzzification, the input variables should be converted to linguistic values to determine the membership degree. Thus, fuzzification is the process of making a crisp quantity fuzzy. The crisp quantity is converted to fuzzy values by recognizing the quantities that are not deterministic, as well as carrying considerable uncertainty. If such uncertainty happens by imprecision, ambiguity or vagueness, then the variable can be represented by a membership function. The outputs of this stage are fuzzy values that can be processed by the inference engine to define the output value. In the proposed scheme, the input crisp values are *Active time*, *Energy* and *Velocity*, as shown in Figure 1.

In this paper, we use the linguistic variables, such as *Very Low*, *Low*, *Medium*, *High* and *Very High*, represented as *VL*, *L*, *M*, *H* and *VH*, respectively, for fuzzy sets. The fuzzy sets *H* and *VH* contain active sensors *S* and *ETR* relay nodes having $\mu_{N}$*(S) = 1* and $\mu_{S}$*(ETR) = 1*, where *μ* represents the degree of membership function and *N* represents the number of sensors in the network. The *M* includes those active sensors *S* having $\mu_{N}\left(S\right)>0$. Similarly, fuzzy set *M* for *ETR* relay nodes contains active sensor nodes having $\mu_{S}\left(ETR\right)>0$. The fuzzy sets *L* and *VL* contain active sensors *S* having $0<\mu_{N}\left(S\right)<1$ and *ETR* relay nodes having $0<\mu_{S}\left(ETR\right)<1$.

**Figure 2:**
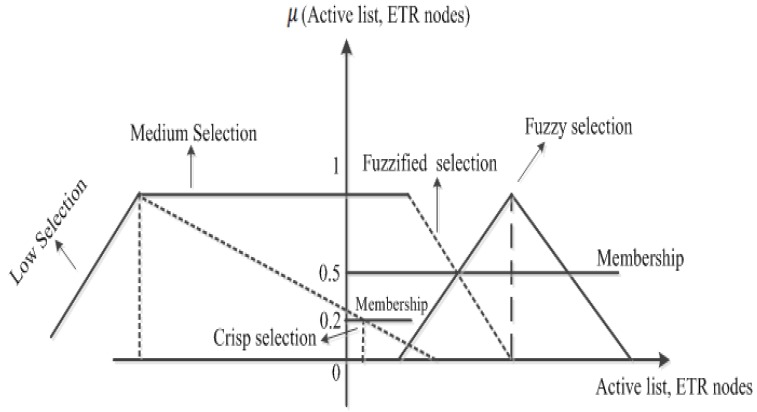
Comparison of fuzzy set, crisp selection and fuzzy selection.

Fuzzy logic deals with the analysis of information by using fuzzy sets. Figure 2 shows the detail of a fuzzy dataset that considers the active/sleep scheduling and *ETR* relay node selection as a crisp and fuzzy selection, respectively. In addition, Figure 2 further shows the comparison between a crisp active/sleep scheduling and an *ETR* selection with a fuzzy set represented as *Low*. Moreover, the figure shows that the crisp selection intersects the fuzzy set at a membership value of 0.2, which is considered as *L*. Similarly, the intersection of the fuzzy set *M*, the fuzzified active set and *ETR* selection occurred at a membership value of 0.5, as shown in Figure 2. Furthermore, it is noticeable that the intersection of the two fuzzy sets is a small triangle whose largest membership value is equal to 0.5, measured as *M*.

In addition to the energy problem, the proposed scheme dynamically discovers the eligible forwarding choices through a fuzzy controller and manages the routing toward the destination. It is important to note that the deadline of a packet is achieved if the required velocity of a packet is met at each relaying node [5]. Hence, the problem of meeting an end-to-end packet deadline is mapped to the local problem of meeting the required velocity of a packet at each relaying node. The proposed fuzzy controller considers the current network conditions to adapt the required velocity of the packet. This implies that the fuzzy controller output can be satisfactory if the required end-to-end velocity of a packet is met at the next hop relaying node.

### 4.2. Inference Engine

The most common way to represent human knowledge in the field of artificial intelligence is to show the knowledge as natural language expressions, such as *IF premise (antecedent), THEN conclusion (consequent)*, where the premise is composed of fuzzy input variables connected by logical functions and consequent result is a fuzzy output variable. The *IF-THEN* rule-based knowledge representation is based on natural language representations and models, which are themselves based on fuzzy sets and fuzzy logic. Thus, the fuzzy engine calculates the value of the output variables based on rules that capture the experts’ knowledge of the controlled system.

In the proposed fuzzy controller, the output variables *Active mode* and *ETR* indicate the scale of change imposed by fuzzy inference rules, as shown in the membership function in Figure 2. In the proposed scheme, the rules indicate that the linguistic variables are changing for the *Active mode* and *ETR* based on the crisp values of input variables, as shown in Table 1. The inference engine performs different calculations in fuzzy logic-based systems. In this paper, the Mamdani inference engine [25] works with the input of a Mamdani fuzzy logic system, that is a crisp value. Thus, the input fuzzy values from the fuzzification stage apply to all fuzzy rules by the inference engine. The control output derived from the combination of input, output membership functions and fuzzy rules are still a fuzzy element. To make fuzzy output accessible to real applications, a defuzzification process is required.

In order to guarantee the network lifetime *α*, the integration of fuzzy logic with the decision process is proposed in this section. In the proposed fuzzy controller scheme, the output variable selection directs the scale of change imposed to the active or sleep mode of a sensor node. The ratio of active time $A_{Time}$ to *α* represents an optimal energy consumption rate. If a sensor node consumes energy with this rate, the node will remain alive until the required lifetime. Let *γ* represent the difference between the optimal and the computed energy consumption rates.

(1) $$\begin{array}{ccc} \gamma & = & \frac{A_{Time}}{\alpha }-\frac{E_{con}}{\beta } \end{array}$$

In Equation (1), the ratio of energy consumption $E_{con}$ and initial energy *β* represents the energy consumption rate of a sensor node in the current and previous round, respectively. If γ≥0, then a sensor node is regarded to have more remaining energy to last for the required guaranteed lifetime *α* at the cost of the optimal energy consumption rate. If γ<0, then a sensor node has already consumed more energy, so it cannot remain alive until *α* with the current energy consumption rate. Thus, the sensor node having γ<0 is expected to go into sleep mode for the next round to save its energy towards *α*.

**Figure 3:**
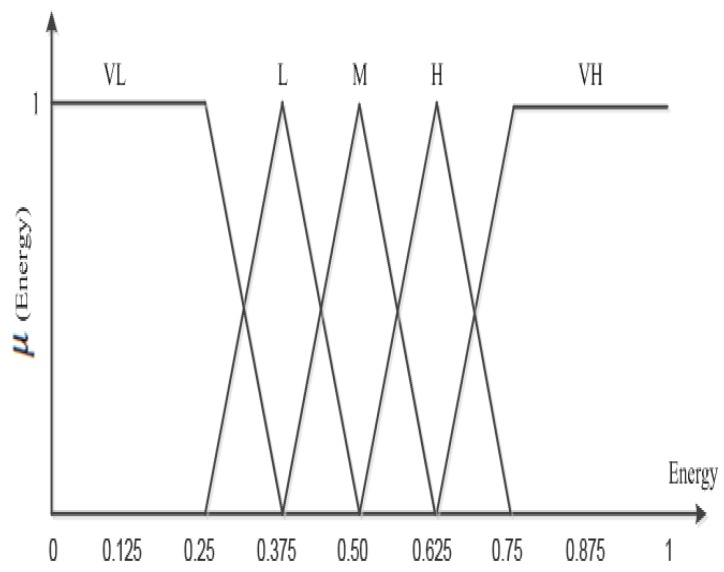
Fuzzy membership function for energy function.

**Figure 4:**
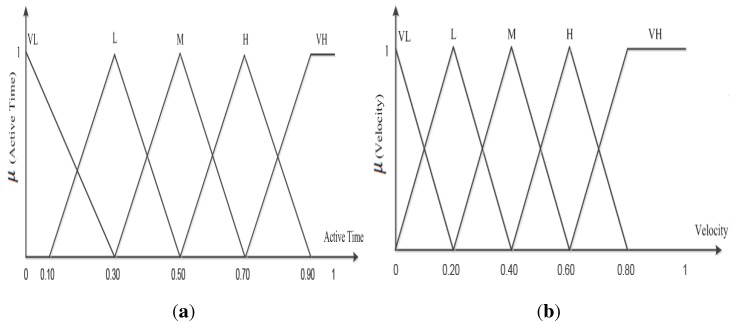
Fuzzy membership function. (**a**) Fuzzy membership function for A*_time_*; (**b**) fuzzy membership function for velocity.

The input descriptors, energy and active time, in a proposed fuzzy control system have the linguistic values *VL*, *L*, *M*, *H* and *VH*, and the membership functions are shown in Figure 3 and Figure 4a. The membership functions indicate that if the energy and active time of a sensor node increases or decreases, the change occurs accordingly. The fuzzy controller output becomes successful if each sensor node *i* has remaining energy greater than zero and unsuccessful if any node *i* exhausts its energy level. The proposed scheme ensures that a fuzzy controller keeps the energy consumption balance among sensor nodes to achieve *α*. Figure 3 and Figure 4a show the degree of membership functions for the inputs and outputs of energy and active time, respectively. To consider each node’s energy and active time, the fuzzy controller assigns a triangular fuzzy value that specifies the range for a given level instead of a particular discrete value. Thus, the triangular fuzzy value provides an easy solution for the described problem with two parameters, *i.e.*, center point and the distance between the extreme points and the center point. To show the membership function for energy *β* and time *α*, the fuzzy controller generates an output value from the set of (0,1).

Due to the diverse assigned tasks, the energy consumption of active nodes in each round may vary. Thus, the active time of a node in a specific round does not indicate the same level of energy consumption in each round. To guarantee a network lifetime *α*, WSNs are largely dependent on the active time of sensor nodes. Therefore, in order to select the active nodes among all nodes, the fuzzy controller considers both remaining energy and active time. The imprecisions or uncertainties in fuzzy logic are controlled by a probability value, which is defined by the membership function.

In addition to a guaranteed network lifetime, real-time delivery can be achieved by reducing the number of hops between source and destination. Consequently, the number of hops can be reduced by expanding transmission power. Thus, the proposed scheme uses transmission power control in the sensor nodes as a grip to reduce end-to-end delay. The fuzzy controller is based on the capability of changing the transmission power of a node to achieve real-time service under a guaranteed lifetime *α*. Thus, the fuzzy controller assigns value to the output from the set of (0, 1), as shown in Figure 4b, to present the membership function for velocity *V* in a pre-configured lifetime *α*.

The proposed fuzzy controller selects the next relay node hop *ETR* to forward real-time packets with increased transmission power in order to achieve the packets’ deadline. On the other hand, the NTR nodes use greedy forwarding and energy-aware routing to transmit data packets towards the sink node for non-real-time packets.

#### 4.2.1. Inference Rules for Active and ETR Node Selection

In this section, we discuss the various elements of uncertainties and decisions made for processes that contain nonrandom uncertainties. Table 1 shows the proposed fuzzy controller rules for the selection of active sensors and *ETR* relay nodes. Furthermore, Table 1 describes the five linguistic variables used as an input for the energy and active time with one possible probabilistic output. We considered a total number of 25 inference rules for each active node and ETR relay node selection, as shown in the following Table 1. Two membership functions, energy and active time, are used for the selection of the active mode. Similarly, two membership functions, energy and velocity, are used for the selection of the *ETR* relay node. As two membership functions contain five respective values, therefore 5×5=25 is the total number of inference rules for each output.

**Table 1:** Inference rules for active and extended transmission range (ETR) node selection.

Rules	Energy	A*_time_*	Velocity	Active Mode	ETR
1	VL	VL	VL	*L*	VL
2	VL	*L*	*L*	VL	VL
3	VL	*M*	*M*	VL	VL
4	VL	*H*	*H*	VL	VL
5	VL	VH	VH	VL	*L*
6	*L*	VL	VL	*M*	VL
7	*L*	*L*	*L*	*M*	VL
8	*L*	*M*	*M*	*L*	*L*
9	*L*	*H*	*H*	*L*	*M*
10	*L*	VH	VH	VL	*H*
11	*M*	VL	VL	VH	VL
12	*M*	*L*	*L*	*H*	VL
13	*M*	*M*	*M*	*M*	*L*
14	*M*	*H*	*H*	*M*	*H*
15	*M*	VH	VH	*L*	*H*
16	*H*	VL	VL	VH	VL
17	*H*	*L*	*L*	VH	*L*
18	*H*	*M*	*M*	*H*	*L*
19	*H*	*H*	*H*	*H*	*H*
20	*H*	VH	VH	*M*	*H*
21	VH	VL	VL	VH	VL
22	VH	*L*	*L*	VH	*L*
23	VH	*M*	*M*	*H*	*M*
24	VH	*H*	*H*	*H*	VH
25	VH	VH	VH	*M*	VH

#### 4.2.2. Energy-Aware Three-Tiered Node Selection

In order to ensure the desired real-time performance and to guarantee the pre-configured lifetime *α*, it is important to define a node selection strategy so that each sensor and relay node can forward its data to multiple relay nodes or directly to the sink. The nodes in the active list are further grouped into sensing, *NTR* and *ETR* nodes to perform different tasks, that is sensing, non-real-time and real-time packets forwarding, respectively.

The key function of the node selection scheme is a three-tier node selection model in the network field. In the first tier, all active sensor nodes are considered as sensing nodes. In the second tier, a minimum number of *NTR* relay nodes are selected to ensure the connectivity of sensor nodes in the network field. A sensing node *i* is said to be covered by the NTR relay node *j* if *i* can transmit its data directly to *j*. Similarly, in the third tier, *ETR* relay nodes are selected to ensure the connectivity of *NTR* nodes and at least one neighbor *ETR* node toward the sink.

Based on the above operations, the objective function of this formulation is to select the minimum number of *NTR* and *ETR* relay nodes in the network field. Let *X =* $\left\{x_{1},x_{1},\cdots ,x_{n}\right\}$ be a set of active sensor nodes with known locations in the network field with communication range r>0. Similarly, let *Y =* $\left\{y_{1},y_{2},\cdots ,y_{n}\right\}$ and *Z =* $\left\{z_{1},z_{2},\cdots ,z_{n}\right\}$ be the sets of *NTR* and *ETR* relay nodes with known locations in the network field, and each *NTR* and *ETR* relay node communication range is $T_{NTR}>r$ and $T_{ETR}>T_{NTR}$, respectively. A set of *NTR* relay nodes, *Y =* $\left\{y_{1},y_{2},\cdots ,y_{n}\right\}$, is considered to be a feasible two-tiered *NTR* relay node selection for *(X, r, T_NTR_)* at any position in the network if:

1. Select node *j* having maximum remaining energy *RE* as the *NTR* relay node from active set *X*;
  $$\underset{\forall i,j\in AL}{arg max}f\left(RE_{j}\right):=\left\{RE_{j}\;|\;\forall i:\;\;RE_{j}>RE_{i}\right\}$$
2. For each sensor node $x_{i}\in X$, there is at least one *NTR* relay node $y_{j}\in Y$, such that $\left|\right|x_{i}\;y_{j}\left|\right|\leq r$;
3. Each *NTR* relay node ∈ *Y* must be connected with at least one other *NTR* relay node toward the sink. Thus, the graph $G=(Y,\;T_{NTR}$*)* is said to be connected if the vertex set of *G* is *V = Y* and the edge set of *G* is;
  $$\begin{array}{c} E=\{\left(y_{i},y_{j}\right)|\;\left(y_{i},y_{j}\right)\in Y,\;||y_{i}\;y_{j}||\leq T_{NTR}\} \end{array}$$

Similar to the two-tier *NTR* relay selection in the previous step, the three-tier *ETR* relay nodes’ set *Z =* $\left\{z_{1},z_{2},\cdots ,z_{n}\right\}$ is considered to be a feasible selection for $\left(Y,T_{NTR},T_{ETR}\right)$ if:

1. Select node *k* having a maximum remaining energy *RE* as the *ETR* relay node from the active set *X*;
  $$\underset{\forall i,k\in AL}{arg max}f\left(RE_{k}\right):=\left\{RE_{k}\;|\;\forall i:\;\;RE_{k}>RE_{i}\right\}$$
2. For each *NTR* relay node $y_{i}\in Y$, there is at least one *ETR* relay node $z_{k}\in Z$, such that $\left|\right|y_{i}\;y_{j}\left|\right|\leq T_{NTR}$;
3. Each *ETR* relay node ∈*Z* must be connected to at least one other *ETR* relay node toward the sink. Thus, the graph $G=(Z,\;T_{ETR}$*)* is said to be connected if the vertex set of G is V = Z and the edge set of G is:
  $$\begin{array}{c} E=\{\left(z_{i},z_{j}\right)|\;\left(z_{i},z_{j}\right)\in Z,\;||z_{i}\;z_{j}||\leq T_{ETR}\} \end{array}$$

### 4.3. Defuzzification

The defuzzification process converts the fuzzy output back to the crisp or classical output to the control objective, just as fuzzification is the conversion of a precise quantity to a fuzzy quantity. The defuzzification phase compiles the required output by using an accumulation function to combine the endorsements of each rule into the single most certain controller output value using the likelihood of each rule being triggered [6]. Among the available three defuzzification techniques, we used the Mamdani centroid technique, which is the most popular technique, as well as being widely utilized in actual applications. The defuzzification phase generates two outputs *Active mode* of the sensor nodes and the *ETR* relay node. The active mode is based on the remaining energy and active time of a sensor node where the selection of the *ETR* relay node to forward real-time packets is based on the node’s remaining energy and velocity. The fuzzy controller uses the following Equations (2)–(4) to calculate the required end-to-end velocity of a packet at each relaying *ETR* node to achieve real-time service.

(2) $$\begin{array}{c} V_{ETR_{i}}\;=\;\frac{D(ETR_{i},Sink)}{R_{i}\left(Tx\right)} \end{array}$$

*D(ETR_i_, Sink)* is the Euclidean distance between the *ETR_i_* and *sink* nodes. *R_i(Tx)_* is the amount of time left before the deadline expires at relay node *i*. The delay occurring between *ETR_i_* and the next hop *ETR* relay node *NH_ETR_i__* is represented as:

(3) $$\begin{array}{ccc} Delay(ETR_{i},NH_{ETR_{i}}) & = & LQ(ETR_{i},NH_{ETR_{i}})\\ & & \;\times (T_{i(Ch)}+T_{i(Tx)}) \end{array}$$

*Delay*(*ETR_i_, NH_ETR_i__*) represents the one-hop delay that a packet faces in the real-time queue, and *LQ* is the ratio of the expected number of transmissions successfully delivered and received between relay node *ETR_i_* and *NH_ETR_i__*. The velocity of expected *NHNH_ETR_i__* is:

(4) $$\begin{array}{ccc} V(NH_{ETR_{i}}) & = & \frac{D(ETR_{i},NH_{ETR_{i}})}{Delay(ETR_{i},NH_{ETR_{i}})} \end{array}$$

$$\begin{array}{ccc} \;where & & \\ & & D(ETR_{i},NH_{ETR_{i}})\leq ETR\;value\\ & & \&\;\;Delay\left(ETR_{i},NH_{ETR_{i}}\right)<R_{i(Tx)} \end{array}$$

To keep the delay at a minimum, the proposed scheme gives high priority to tight deadline packets in the real-time queue. *RT* is initially set to be the end-to-end deadline at the source node and decremented at each hop to account for queuing, contention and transmission delays [4]. The power-aware transmission scheme not only improves link quality, but also reduces the number of transmissions needed to deliver a real-time packet.

## 5. Performance Evaluation

In order to evaluate the performance of the proposed algorithm, we conducted simulations through ns-2.34 with the parameter values shown in Table 2. We adopted the IEEE 802.15.4 specification model as a reference to carry out efforts in the battery module. We conducted extensive simulation scenarios of the proposed algorithm represented as fuzzy in the graphs. We compared the results for guaranteed pre-configured lifetime *α* and real-time communication with FML [12] and our previous scheme EPGLT [5].

**Table 2:** Simulation parameters. NTR, normal transmission range.

Sensing field dimensions	400 × 400 m
Number of sensor nodes	100
Node placement	Random
Initial energy of each node	300 J
Sensing power	0.350 W
Listening power	0.320 W
Transmitting power	NTR = 0.550 W, ETR = 0.700 W
Receiving power	0.400 W
Sleeping power	0.001 W
Sensing frequency	0.1 Hz
Radio transmission range	(60,130) m
Packet size	50 bytes
Maximum rounds	10

### 5.1. Analysis for Guaranteed Lifetime

**Figure 5:**
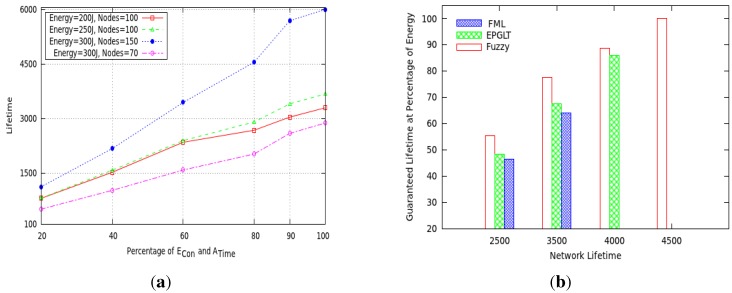
Various values of *α* to guarantee. (**a**) Network lifetime; (**b**) guaranteed lifetime at the % of energy.

We validated the performance of a pre-configured network lifetime using the fuzzy-based algorithm, discussed above, with four different measurement values of *α*. To see the significance of the pre-configured network lifetime, we compared four cases on a network with different parameter settings for each period of *α*. For each of these four cases, the pre-configured network lifetime *α* is calculated *a priori* and is plotted in Figure 5a,b. In the test instances, we *a priori* determined four values of *α* ∈ (2650 s, 3500 s, 6000 s, 2800 s) based on the parameter settings, such as ({Nodes = 100, Energy = 200 J}, {Nodes = 100, Energy = 250 J}, {Nodes = 150, Energy = 300 J}, {Nodes = 70, Energy = 300 J}), respectively. In all four test scenarios, the energy consumption and active time follow a uniform distribution toward the pre-configured lifetime, as shown in Figure 5a. The proposed fuzzy controller utilized the energy and sleep schedule among nodes in an efficient way and guaranteed all of the *a priori* determined lifetimes. In each round, the fuzzy controller selects active nodes on defined inference rules to keep a level of energy balance among all sensor nodes in the network. The proposed fuzzy controller guarantees the first test case pre-configured lifetime *α* = 2650 s at a rate of less than 90% of the total energy consumption, as shown in Figure 5a.

Moreover, the proposed scheme compared four *a priori* determined lifetimes *α* ∈ (2500 s, 3500 s, 4000 s, 4500 s) on a network with the same parameter settings against the FML and EPGLT protocols in Figure 5b. The proposed fuzzy controller achieved the pre-configured lifetime at the rate of 55%, 77.77%, 89% and 100% total energy consumption, respectively. As shown in Figure 5b, the FML and EPGLT protocols did not consume their total energy; however, some of the nodes in the network drain out their energies at an early time due to unbalanced energy consumption. This fact brings the network lifetime to an end in both FML and EPGLT protocols.

**Figure 6:**
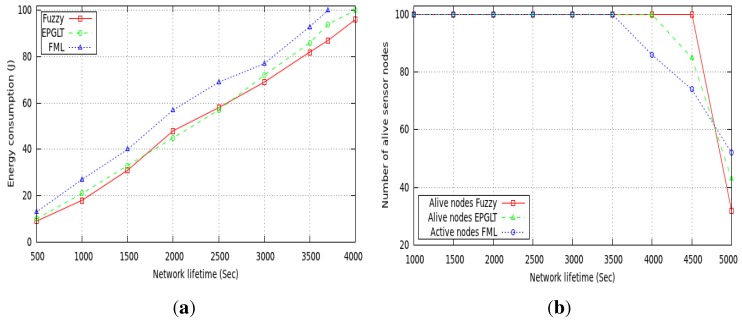
Network lifetime. (**a**) Guaranteed lifetime; (**b**) working and live nodes in each round.

For the rest of the analysis, we set the pre-configured network lifetime at *α* = 4000 s according to the application demand. The total energy consumption among active nodes in each round is illustrated in Figure 6a, where a sensor is transmitting packets to its neighbor relay node toward the sink. In each round, the proposed fuzzy model selects nodes among the active/sleep modes in an energy-efficient way to guarantee the required network lifetime *α* = 4000 s. As shown in Figure 6a, our fuzzy scheme performs better and guarantees the pre-configured lifetime *α* = 4000 s at the total cost of less than 100% of energy consumption. The fuzzy routing algorithm, FML, is directly dependent on the transmission radius of the sensor node to route data. Thus, each node is discovering more neighbors in its radius, which consumes more energy. Furthermore, as the transmission radii increase, more energy is required to discover the maximum number of neighbor nodes. On the other hand, EPGLT amalgamates the energy-aware routing with power-aware transmission to guarantee the pre-configured network lifetime. The drawback of both schemes is the unbalanced energy consumption after reaching the pre-configured lifetime.

Figure 6b shows the effect of adjusting transmission range and active/sleep schedules on the number of alive nodes over time. When a node continues to perform its duty without the optimized selection of transmission range and active/sleep schedules, the number of alive nodes in the network decreases rapidly, as the plot of FML and EPGLT shows in Figure 6b. On the other hand, the proposed fuzzy controller selects the nodes for the active mode in each round, which are further categorized through the energy-aware three-tiered node selection. Thus, the transmission range and the active/sleep schedules among nodes are selected by defined inference rules that guarantee the pre-configured network lifetime; node failure occurred after the configured lifetime, as shown in the Figure 6b. Furthermore, the figure shows that the nodes start dying after 4000 s in the proposed scheme, where the nodes in FML and EPGLT drain out their energies before the configured time, which brings the network lifetime to an end. Figure 6b shows that the first node died in the FML protocol at 3500 s and in EPGLT at 4000 s, whereas in the fuzzy scheme the first node died after 4500 s.

**Figure 7:**
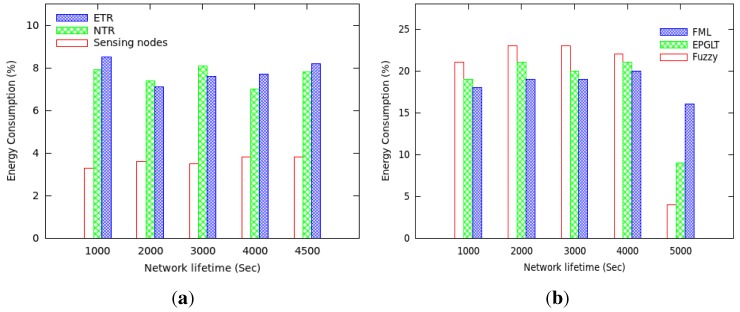
Network lifetime *vs*. energy consumption. (**a**) Energy consumption among nodes; (**b**) energy consumption in rounds.

The transmission range of sensor nodes can affect the network lifetime. Our scheme is dealing with different transmission ranges for real-time packets; therefore, we evaluate the performance of the proposed fuzzy model under different settings of transmission range. Figure 7a,b shows the results of our proposed approach with a view toward studying the effect of incorporating the energy consumption objective in the guaranteed lifetime process. Recall that the proposed scheme assigns different transmission range relay nodes to routes in real-time and non-real-time data packets. In order to study the energy consumption of the proposed protocol on each active node in a three-tiered model, we examined the different set of nodes over the network lifetime and plotted the results in Figure 7a. The plots showed that the energy consumption in NTR and ETR relay nodes are nearly the same due to the energy and active time-aware schemes applied by the fuzzy controller through fuzzy inference rules in the network to achieve the guaranteed lifetime. The set of active sensors responsible for sensing the network field consumes energy almost at the same rate in all rounds.

Figure 7b shows the average energy consumption of all nodes at different rounds under a uniform data generation pattern. The higher energy consumption at each round is observed in the proposed scheme more than the other two, but is well balanced in most of the rounds. Since FML and EPGLT do not consider a balanced energy scheme in the routing decision, more nodes have enough residual energy when the first node’s energy is drained out, as shown in Figure 7b.

### 5.2. Real-Time Analysis

**Figure 8:**
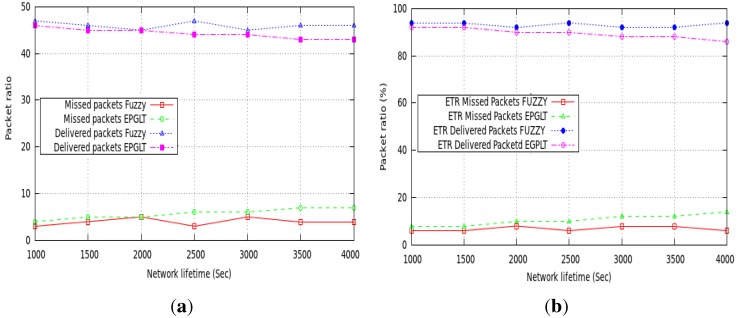
Packet delivery ratio. (**a**) Network lifetime; (**b**) packet ratio.

Performance comparison of real-time data packets in a guaranteed network lifetime between the proposed scheme and EPGLT is discussed in this section. To evaluate the performance of packet delivery in Figure 8a, the simulation is performed on the average ratio of successful and missed end-to-end packets toward the sink. The proposed protocol selects the next hop relaying node through the fuzzy controller and adjusts extended transmission on real-time packets to achieve the end-to-end deadline in the network. To see the significance of the proposed scheme, we assign different deadline values ∈ (150 s, 200 s, 250 s, 300 s) to real-time packets. We run the simulation 100 times for different settings of real-time packet deadlines and plotted the average results in Figure 8a,b. Figure 8a further demonstrates both the fuzzy and EPGLT schemes using multi-hop routing techniques to route data packets to achieve soft real-time communication. Even though EPGLT is based on a mathematical model to select the next hop relaying node, its delivery ratio is lower than ours, since it assigns the same transmission power to forward a real-time packet toward the sink.

It is discerned from Figure 8a that the proposed scheme improves the delivery ratio within the deadline for traffic of different priority levels as compared to our previous scheme and EPGLT. The proposed fuzzy controller scheme successfully forwards packets to the sink with a steady on-time delivery rate throughout the network lifetime. The proposed scheme also shows better performance on data packets’ missed deadlines. The simulation results are based on 50 real-time packets generated and forwarded to the sink node in each round. Out of 50 real-time packets, in the Fuzzy scheme, less than five packets missed their deadlines in each round. On the other hand, the ratio of missed packets in EPGLT is less than 10 in each round. The ratio of successful packets reaching the sink node within the deadline is 6% higher than EPGLT. Similarly, the ratio of packets that missed their deadline by reaching the sink node is 11% less than EPGLT.

Furthermore, Figure 8b shows the end-to-end packet delivery ratio of guaranteed and missed packets using the *ETR* and *NTR* relay nodes for the whole communication during the simulation time. The simulation analysis is done for both techniques separately to check the delivery ratio in more detail. The simulation results showed that the fuzzy controller-based *ETR* relay nodes’ selection performed very well and delivered more than 93% of packets successfully.

## 6. Conclusions

We proposed a new guaranteed pre-configured lifetime scheme with real-time packet support in WSNs. We proved that fuzzy logic is a powerful and accurate mechanism that can be applied successfully to any emergency WSN applications for effective and efficient decisions on a real-time basis. Compared to other models having crisp values, fuzzy logic maintains a high accuracy level and a balanced energy consumption among sensor nodes in the network field. This helps the proposed scheme to adjust each node’s energy consumption approximately at the optimal energy consumption rate to achieve the objective. Furthermore, the design of the fuzzy-logic system is simple and easy, which allows users/applications to define different variables, sets and rules, depending on each particular environment and the sensor features. Our work utilized the rich energy sensor nodes for real-time service and proved that all sensors utilized their maximum energy level by reaching a pre-configured lifetime.
